# Do norms unintentionally increase stereotypical expressions? A randomised controlled trial

**DOI:** 10.1111/medu.14712

**Published:** 2021-12-26

**Authors:** Chantal E. E. van Andel, Marise P. Born, Walter W. van den Broek, Karen M. Stegers‐Jager

**Affiliations:** ^1^ Institute of Medical Education Research Rotterdam Erasmus MC Rotterdam The Netherlands; ^2^ Department of Psychology Erasmus University Rotterdam Rotterdam The Netherlands; ^3^ Optentia and Faculty of Economic and Management Sciences North‐West University Potchefstroom South Africa

## Abstract

**Introduction:**

Implicit biases of health professionals could cause biased judgements. Many anti‐bias interventions seem to be ineffective, and some even counterproductive. People tend to be compliant to standards describing what the majority of people finds or does, and this could cause people to think in a stereotype‐consistent manner. This study examines whether descriptive social norms such as ‘the majority of people have stereotypes’ (majority message), as often stated in interventions, actually increase people's stereotypes. To examine the effect of descriptive social norms (Hypothesis 1) and the effect of individual perceptions and preferences (Hypothesis 2a and 2b) on stereotypical expressions towards medical students.

**Methods:**

First, we determined which ethic stereotypes regarding medical students prevail in Dutch medical education (*N* = 52). Next, two similar randomised controlled trials, both with teachers and students, were carried out (*N* = 158 and *N* = 123, respectively), one with an East Asian student picture (ethnic minority) and one with a native Dutch student picture (ethnic majority). Participants were randomly assigned to either a majority‐message, minority‐message or no‐message condition, and rated the presented minority or majority picture on specific stereotypical features. Subsequently, participants described a typical day of that same student's life. These descriptions were rated for stereotypicality by two independent raters, who were blind for condition and stimulus. Inclusive work environment (IWC) and social dominance orientation (SDO) of participants were measured as indicators of individual perceptions and preferences.

**Results:**

Stereotypes were expressed towards both picture stimuli, yet message condition did not affect stereotypical expressions. SDO positively related to stereotypical expressions towards the East Asian student, whereas IWC positively related to stereotypical expressions towards the native Dutch student.

**Conclusion:**

Interventions do not unintentionally increase stereotypes by communicating what the majority of people thinks or does. Individual perceptions and preferences are predictive of stereotypes, whereas descriptive social norms are not.

## Abstract 2

The authors empirically test concerns about anti‐bias interventions unintentionally increasing stereotyped expressions, additionally reporting individual beliefs to be more predictive of such expressions than context.

## 1 INTRODUCTION

Implicit bias in health professionals could cause inaccurate evaluations of students,^1^, ^2^ as well as inaccurate treatments of patients from minority groups.^3^, ^4^, ^5^ Implicit bias is typically used to refer to implicit prejudices and stereotypes that could result in biased behaviours.^6^, ^7^ A recent systematic review in real world contexts has shown that many interventions to reduce implicit bias have no effect, especially when it comes to the long term.^7^ Moreover, interventions could even be counterproductive^8^, ^9^ and could create illusions of fairness that cause majority group members to become less sensitive to recognising discrimination against people from minority groups.^10^ This study examines whether descriptive social norms, for example communicating a high prevalence of stereotypes, could actually be counterproductive. Therefore, this study tests whether a majority‐norm message such as ‘the majority of people have stereotypes’, could actually increase expressions of stereotypes towards medical students from either a stigmatised (ethnic minority) or non‐stigmatised (ethnic majority) group.

People tend to be compliant to descriptive social norms, because they are likely to adhere to standards describing what the majority of people finds or does.^11^ More specifically, the social influence of norms can cause people to value diversity if everyone else in an organisation seems to value diversity, but it can also cause people to be prejudiced if other people seem to be prejudiced.^9^ Indeed, research has shown powerful effects of norms on people's prejudice not only in comments in online settings and video games,^12^, ^13^ but also in social interactions.^14^ In order to stimulate people to reduce their bias, it is therefore important to recognise the role of social context.^15^ Repeatedly communicating a high prevalence of stereotypes, in for example, anti‐bias interventions, could cause normalisation. This normalisation process might actually exacerbate bias rather than challenge it,^16^ because ‘if everyone is biased, it is OK if I am too’.^17^

This research experimentally tests whether messages displaying different descriptive social norms, that is, majority‐messages such as ‘the majority of people have stereotypes’ versus minority‐messages such as ‘the minority of people have stereotypes’ or no message, have different effects on medical teachers and students' stereotypical expressions. Our first hypothesis is that the majority‐norm will increase people's stereotypical expressions. This research has a similar procedure as an earlier study in psychology that used women, elderly people and obese people as stigmatised groups.^16^ It adds novelty to the literature because it applies research with an ecological valid sample in a realistic setting, that is, a healthcare setting with systematic inequalities in experience and outcomes based on people's social group memberships.^18^, ^19^, ^20^ It also uses a different stigmatised minority, and deliberately adds the non‐stigmatised majority group as a stimulus, as stereotypes could also be positive, and contribute to systematic differences in power and privilege as such.^21^

Additionally, assuming that behaviour results from an individual in a context, this study examines whether individual perceptions and preferences could be more or less predictive of stereotypes than the context. Therefore, two additional measures are taken into account. First, the extent to which people believe that they actually work or study in an inclusive work environment (IWC) is measured,^22^ as this belief might influence the perceived norm of whether or not the majority discriminates. It is thus expected that the higher someone's IWC, the lower the stereotypical expressions (Hypothesis 2a). Second, social dominance orientation (SDO) is measured^23^ as an individual preference for group based hierarchy and inequality has been linked to the tendency to prejudice.^13^, ^24^ Individuals higher in SDO endorse domination of one group over other groups in a society and desire to maintain or even increase differences between social groups.^25^ It is thus expected that the higher someone's SDO, the higher one's stereotypical expressions (Hypothesis 2b).

## 2 METHODS

### 2.1 Research design

**FIGURE 1:**
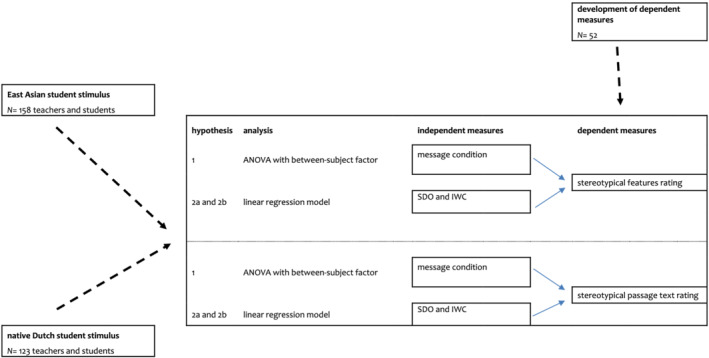
An overview of the study flow: development of dependent measures, the two student stimuli with both their own dataset, and the analyses for each of the hypotheses. IWC, inclusive work environment; SDO, social dominance orientation [Color figure can be viewed at wileyonlinelibrary.com]

The first phase of the study concerned the development of the dependent measures (see Data S1). The second phase, that is the current research, concerns a prospective double‐blind randomised controlled trial with one between‐subject factor with three conditions for descriptive norms (*majority‐message*, *minority‐message* or *no message condition*) and two dependent measures that both indicate stereotypical expressions. This trial is carried out twice, first with a stigmatised (ethnic minority) stimulus, second with a non‐stigmatised (ethnic majority) stimulus (see Figure 1).

### 2.2 Participants and procedure

For the two trials in total, participants were 95 teachers, 82 bachelor students and 104 master students who worked or studied at Erasmus MC Medical School in Rotterdam, the Netherlands. This school has a relatively large number of ethnic minority students (~30%). Teachers were considered an ecologically valid group to include, given that the workplace‐based assessments of students could be impacted by their susceptibility to stereotypes.^20^ Students were also included, because the effect of descriptive norms on stereotypes was expected to exist regardless of age^16^ and because they are educated to become doctors, preferably unaffected by implicit biases. Participants were actively recruited, via e‐mail, via online lectures in Zoom or in person. Participants were asked to complete an online survey in Qualtrics in which they had to give ratings to and write a short passage about a student who was displayed in one picture. Next, participants indicated their levels of SDO and IWC, followed by demographical questions. Participants were informed that they took part in a ‘study that investigated person‐perceptions, for instance the ability of doctors to estimate details of life‐events on the basis of visual information’. This information functioned as our cover story, meaning that participants were unaware of the fact that the study measured stereotypical expressions and that they were experimentally manipulated with different conditions, each displaying a different descriptive norm message. The study took 10 min of their time. No compensation was offered.

### 2.3 Picture stimuli

An East Asian student functioned as the stigmatised (ethnic minority) student and a native Dutch student functioned as the non‐stigmatised (ethnic majority) student. Female students were deliberately chosen for both picture stimuli, as female students are the largest gender group in Dutch medical schools and to exclude unique effects of gender in ratings. Both students were dressed in a white coat, with neutral facial expressions and their hairs tied, in front of a neutral background (see Data S3). Each participant only saw one stimulus.

### 2.4 Experimental manipulation

Participants were randomly assigned to one of three conditions: a majority‐message condition (The vast majority of people have) or minority‐message condition (Very few people have) ‘stereotypical preconceptions and their impressions and evaluations of others are consistently biased by these stereotypic preconceptions. You should actively try to avoid thinking about others in such a manner’ or no message condition. We deliberately chose to include the admonition ‘try to avoid stereotyping’, because it resembles the real world in which people are increasingly told that they should not stereotype, and it did not affect the findings in the study that was similar to ours.^16^ Participants read the message right before scoring each of the dependent measures.

### 2.5 Dependent measures

Data of attendees (*N* = 52) of the Dutch Society for Medical Education conference in November 2019 were used for the development of our dependent measures, that is, stereotypical features rating and stereotypical passage text ratings for both picture stimuli (see Data S1). First, for both trials, participants were asked to rate the student in the picture on stereotypical features. In the randomised controlled trial that involved the East Asian student, participants rated the student on the features *assertiveness*, *communication skills*, *intelligence* and *knowledge of Dutch hospital culture* (*ω* = 0.67). The randomised controlled trial that involved the native Dutch student, asked participants to rate student on the features *ambitiousness*, *eloquence*, *competence*, *diligence* and *intelligence* (*ω* = 0.84). All stereotypical features, for both stimuli, were scored on a scale from 1 (*not at all*) to 7 (*very much*). To ensure that higher ratings implied higher stereotypical expressions, the four items for the East Asian student were reversed (see also Data S1). An average score for the features rating was computed for both stimuli.

Second, for both trials, participants were asked to ‘write a description of a typical day in the life of the student displayed in the picture’. Participants' answers were then coded by two other raters who also had experience with qualitative data coding, and were blind to message conditions, and were blind to stimuli. Raters were instructed to independently code the passages of texts from both trials on the basis of (a) preconceived notions, (b) stereotypes of native Dutch students and (c) stereotypes of East Asian students,^16^ on a three‐point scale (1 = *low*, 2 = *medium*, 3 = *high)*. Preconceived notions were defined as all subjective inferences that were not stereotypes per se. Examples include the following: the student snoozes her alarm in the morning; has a boyfriend; watches Netflix. To code the level of stereotypicality, raters compared answers with the complete lists of stereotypical features as derived in the first phase of the study (see Data S2). Raters were first trained on the rating system and rated some passages along with the main author. After completion, initial rater agreement^26^ was 67.8%, 70.7% and 66.3% for preconceived notions, for East Asian student stereotypes, and for native Dutch student stereotypes, respectively. Areas of strong disagreement, that is ratings that differed two points on a three‐point scale between both raters (10.32% of all ratings), were reconsidered and discussed, and consensus was sought. After consensus, intraclass correlation coefficients (ICCs) and Cohen's Kappa (κ) were used to measure the consistency among multiple ordinal observations of two raters.^27^ The average ICC was 0.88 (95% confidence interval [CI] [0.85–0.90], *p* < 0.01), and κ = 0.57, (*p* < 0.01) for the East Asian stimulus, and 0.83 (95% CI [0.78–0.86], *p* < 0.01) and κ = 0.48, (*p* < 0.01) for the native Dutch stimulus. Hence, moderate to good inter rater reliabilities were found,^28^ and the ratings for each stimulus were averaged.

### 2.6 Participant demographics and independent measures

Participants' gender, age, (parents') country of birth and function type (teacher or student) were reported. IWC was measured with a validated 6‐item Dutch scale (*ω* = 0.81 in this study),^22^ with answer scores ranging from 1 (*totally disagree*), to 4 (*totally agree*). Example items are ‘At work, I can openly express my opinion without having to fear negative consequences’ and ‘My organization has a work environment in which discrimination does not occur’. For students, ‘work’ was replaced with ‘study’ in all items. SDO was measured with a validated 8‐item scale,^23^ with answer scores ranging from 1 (*totally disagree*), to 7 (*totally agree*). The scale was translated using a back‐translation procedure.^29^ Four items measure social group dominance (SDO‐D), and four items measure social group inequality (SDO‐E). Example items are ‘An ideal society requires some groups to be on top and others to be on the bottom’ (SDO‐D) and ‘We should do what we can to equalize conditions for different groups’ (reversed item for SDO‐E). Treating SDO as one factor fitted the data best,^23^ and hence, one average SDO score was computed (*ω* = 0.78 in this study).

### 2.7 Ethics statement

Participation in this study was voluntary, and written informed consent was obtained from all participants. The informed consent involved a non‐disclosure, as uncovering the real research aims would plausibly affect the results. Participants were debriefed with the real research aims after completion of the survey. They were allowed to withdraw their data until 2 weeks after the debriefing. The data were pseudo‐anonymous, as the researchers had to enable participants to withdraw their (otherwise anonymous) data. For this aim, participants created their own unique code at the start of the study. No participants withdrew their data. Ethical permission was approved by the Medical Research Ethics Committee (METC) at Erasmus MC Medical School (dossier number MEC‐2020‐0123).

### 2.8 Statistical analysis

For both stimuli, and hence for both trials, Hypothesis 1 was tested with an analysis of variance (ANOVA) with message condition as a between‐subjects factor, on the two dependent measures: features rating and passage text ratings. Hypotheses 2a and 2b were tested with a linear regression model, with SDO and IWC as independent measures of the two dependent measures: features rating and passage text rating (see Figure 1 for an overview of the study flow). Our minimum recruitment target was set to 128 participants in total for each stimulus, in order to detect a medium effect size (*f* = .25) with three groups, an error probability of *α* = 0.05, and power β = 0.80, as calculated with G*Power software.^30^

## 3 RESULTS

### 3.1 East Asian student

First, the results for the randomised controlled trial that involved the East Asian student are presented. Participants were 37 undergraduate, 37 graduate students and 84 teachers. The total sample consisted of *N* = 158 participants (62.9% female and 85.0% Dutch), with a mean age of *M* = 33.30, *SD* = 13.67, ranging from 19 to 65 years old. They had been randomly assigned to a majority message (*N* = 56), a minority message (*N* = 43) or no message (*N* = 59), see Table 1.

**TABLE 1:** Descriptive statistics across experimental conditions

		Total		Majority message		Minority message		No message	
		N	*M* (*SD*)	N	*M* (*SD*)	N	*M* (*SD*)	N	*M* (*SD*)
East Asian student stimulus	Features rating	158	3.05 (0.69)	56	3.07 (0.63)	43	2.97 (0.66)	59	3.10 (0.78)
	Preconceived notions	154	2.15 (0.75)	55	2.13 (0.75)	40	2.00 (0.74)	59	2.26 (0.76)
	Passage text ratings	154	1.64 (0.75)	55	1.60 (0.73)	40	1.51 (0.76)	59	1.75 (0.76)
	Social dominance orientation	147	2.66 (0.93)	54	2.66 (0.93)	38	2.30 (0.84)	55	2.47 (1.01)
	Inclusive work culture	152	2.89 (0.58)	55	2.80 (0.64)	40	2.95 (0.53)	57	2.92 (0.55)
Native Dutch student stimulus	Features rating	123	5.32 (0.67)	47	5.21 (0.75)	42	5.29 (0.66)	34	5.49 (0.56)
	Preconceived notions	122	2.09 (079)	46	2.13 (0.79)	42	1.98 (0.79)	34	2.16 (0.81)
	Passage text ratings	122	1.58 (0.67)	46	1.59 (0.64)	42	1.46 (0.64)	34	1.71 (0.76)
	Social dominance orientation	116	2.70 (0.51)	44	2.72 (0.51)	41	2.76 (0.41)	31	2.60 (0.60)
	Inclusive work culture	109	2.72 (0.96)	43	2.52 (0.93)	36	3.02 (0.87)	30	2.72 (0.96)

#### 3.1.1 Features rating

Of East Asian features ratings, 39.1% was between scores 5 and 7, which is considered a high rating of negative stereotypes, given the fact that the scale is between 1 (*low*) and 7 (*high*). Findings showed that message condition did not have an influence on East Asian features rating (*F*_[2, 155]_ = 0.39, *p* = 0.68), therefore, no support for Hypothesis 1 was found (see Table 1). SDO was significantly positively related to East Asian features rating (*B* = 0.17, *t*_[146]_ = 2.80, *p* < 0.01), implying that higher levels of SDO lead to more negative stereotypical features ratings. This finding remained even when controlling for type of respondent (teacher versus student). IWC was unrelated to East Asian features rating (*B* = 0.00, *t*_[146]_ = .02, *p* = 0.98). Hence, support was found for Hypothesis 2a, but not for 2b.

#### 3.1.2 Passage text ratings

Three examples of passage texts that were written for the East Asian student are reported below. The first two examples were scored high on perceived notions and high on stereotypical content, whereas the third example scored low on both.

‘This clerk tries to do her best and tries to be prepared well for this clerkship. Unfortunately, something went wrong this day, because she could not answer an important question from a supervisor. This made her sad and worried about her functioning.’

‘Ambitious student. She wants others to have good impressions of her (smart, hardworking, caring for patients, good colleague) in this clerkship internal medicine, because she wants to specialize in this field in the future. She is cautious in order to prevent negative impressions, but she could mess her chances up while doing this. She is insecure, anxious to fulfil tasks she cannot handle. She raises the bar high for herself and others.’

‘This student goes to her clerkship, begins with an information transfer and participates in the outpatient clinic with a resident in order to close the day with yet another information transfer.’

Of passage texts, 31.2% was rated high on preconceived notions, and 15.6% was rated high on stereotypical content. Findings showed that message condition did not have an influence on East Asian stereotypical passage text ratings (*F*_[2, 151]_ = 1.21, *p* = 0.30); therefore, no support for Hypothesis 1 was found (see Table 1). Further, message condition did not have an effect on preconceived notions that were rated for the East Asian student, (*F*_[2, 151]_ = 1.48, *p* = 0.23). Both SDO (*B* = 0.03, *t*_[142]_ = 0.48, *p* = 0.63) and IWC (*B* = −0.09, *t*_[142]_ = −0.76, *p* = 0.45) were unrelated to East Asian stereotypical passage text ratings. Hence, no support for Hypotheses 2a and 2b was found.

### 3.2 Native Dutch student

Second, the results for the randomised controlled trial that involved the native Dutch student are presented. Participants were 45 undergraduate and 67 graduate students, and 11 teachers. The total sample consisted of *N* = 123 participants (68.6% female and 76.0% native Dutch), with a mean age of *M* = 24.47, *SD* = 7.25, ranging from 18 to 66 years old. They were randomly assigned to a majority‐message (*N* = 47), a minority‐message (*N* = 42) or no message (*N* = 34).

#### 3.2.1 Features rating

Of native Dutch features ratings, 63.4% was between scores 5 and 7, which is considered a high rating of positive stereotypes, given the fact that the scale is between 1 (=*low*) and 7 (=*high*). Findings showed that message condition did not have an influence on native Dutch features rating (*F*_[2, 120]_ = 1.68, *p* = 0.19), therefore, no support for hypothesis 1 was found (see Table 1). Both SDO (*B* = .01, *t*_[108]_ = 0.09, *p* = 0.93) and IWC (*B* = 0.21, *t*_[108]_ = 1.70, *p* = 0.09) were unrelated to native Dutch features rating. Hence, no support for Hypotheses 2a and 2b was found.

#### 3.2.2 Passage text ratings

Three examples of passage texts that were written for the native Dutch student are reported below. The first two examples were scored high on perceived notions and high on stereotypical content, whereas the third example scored low on both.

‘Looks capable, will be taken seriously during her clerkship. She will be allowed to work independently in short term’.

‘This clerk looks good and healthy. She probably does sport and eats healthy food on a typical day. This clerk looks confident, maybe a bit closed, which creates the impression that she is not lazy or passive.’

‘This clerk wakes up early in the morning in order to go to the hospital. She takes on her white coat and joins/receives guidance from one or more doctors throughout the day’.

Of passage texts, 30.3% was rated high on preconceived notions, and 10.7% was rated high on stereotypical content. Findings showed that message condition did not have an influence on native Dutch stereotypical passage text ratings (*F*_[2, 119]_ = 1.22, *p* = 0.30); therefore, no support for Hypothesis 1 was found (see Table 1). Further, message condition did not have an effect on preconceived notions that were rated for the native Dutch student stimulus, (*F*_[1, 119]_ = 0.62, *p* = 0.54). Therefore, no support for Hypothesis 1 was found. SDO (*B* = −0.01, *t*_[107]_ = −0.19, *p* = 0.85) was unrelated to native Dutch stereotypical passage text ratings, yet IWC (*B* = 0.26, *t*_[107]_ = 2.04, *p* = 0.04) was positively related, implying that higher levels of IWC lead to more positive stereotypical passage text ratings. This finding remained even when controlling for type of respondent (teacher versus student). Hence, no support for Hypotheses 2a and 2b was found.

## 4 DISCUSSION

This study has sought to examine the effect of descriptive social norms on expressions of stereotypes towards a minority and majority medical student. Stereotypical expressions were independent of exposure to messages depicting what the majority or minority of people thinks or does. Although the study did not provide support for our first hypothesis, participants yielded stereotypical content and expressed preconceived notions towards both stimuli, even when asked to not stereotype. Also, people higher in SDO were more likely to express stereotypes towards a minority student. Furthermore, people who perceive to study/work in an inclusive environment express more positive stereotypes regarding a majority student. Hence, this study showed that individual preferences and perceptions (i.e., SDO and IWC) were predictive of stereotypical expressions, whereas the context (i.e., descriptive social norms) did not.

The present study could not replicate the findings related to social norms on stereotypes of an earlier study with other stigmatised groups.^16^ An explanation for the absence of an effect could be that the content of our message was referring to people in general rather than doctors. Perhaps the message would have had more impact if the message specified that the ‘majority of doctors’ rather than the ‘majority of people’ have stereotypes. Indeed, level of group identification determines the extent to which people are influenced by information of others' beliefs.^31^ Future research could examine whether communicating norms in terms of moral ideals (e.g., ‘*this organization creates inclusion among all members*’) stimulates more favourable attitudes of majority members towards diversity and equality in a healthcare setting, as compared with moral obligations (‘this organization does not discriminate’).^9^, ^32^ This study, however, specifically depicted messages regarding the prevalence of stereotypes among people in general, like the earlier study,^16^ as many anti‐bias interventions refer to stereotyping as being a universal phenomenon, and not as something specific for doctors. Another potential explanation as to why we have not been able to replicate the findings of a previous study^16^ is the use of another (non)stigmatised group, other feature ratings, and raters who were also blind to stimuli. Unlike the earlier study, we were able to blind the raters, because our study involved a stigmatised and a non‐stigmatised stimulus.

Furthermore, our findings indicated that SDO was positively related to East Asian features rating, but unrelated to the native Dutch features rating. This is fully consistent with literature showing that SDO could especially be predictive of prejudice towards groups that are socially subordinate, or low in status and power.^13^, ^25^ Research has shown that individuals with higher SDO are less likely to hire non‐native candidates^25^ and are more resistant to intercultural dialogues.^33^ A practical implication would be to identify persons with high levels of SDO and encourage them to reduce their preference by means of helping groups, as helping behaviours can decrease (perceptions of) power dynamics between social groups.^34^ Furthermore, we found that IWC was positively related to native Dutch stereotypical passage text ratings. This is contrary to our hypothesis, as we expected that perceptions of inclusion would lead to less negative stereotypes towards minority students, rather than to more positive stereotypes towards majority students. On the contrary, if the work culture communicates a norm of inclusion, but majority students are mainly the ones who actually get included, than perhaps this finding is not surprising. Future research is urged to replicate this finding.

This study yielded stereotypical content towards both stimuli. Evidence for this was that even names, personality characteristics, skills, interests, private issues, and so forth were all subjectively inferred on the basis of a single picture. However, no effect for message condition on stereotypical expressions was found. Whereas our study focused on the ‘normalisation’ of stereotypes, future researchers may want to focus on the normalisation of implicit bias. Bias if often framed as implicit or unconscious, yet it is unsure whether this framing is legitimate.^35^, ^36^ Meanwhile, framing bias as implicit can have severe negative consequences as it reduces people's motivation, accountability and responsibility regarding bias reduction.^37^, ^38^ Also, it could pave the way for ignorance,^39^, ^40^, ^41^ and undermine perceptions of the severity of discrimination.^42^ Hence, how we frame our messages in interventions (‘your bias is unconscious and uncontrolled’ versus ‘your bias can be reduced’) could have implications for how well we succeed in reducing bias and promoting diversity.

A strength of this study was that the first phase of the study specifically determined which stereotypes prevail in medical education and created our dependent measures. Another strength was its' experimental design, the use of blind raters, and the inclusion of two additional independent measures. Furthermore, we included teachers as well as students, which increases the generalisability of our findings. A limitation, however, is that we did not have enough statistical power to test the assumption that teachers and students are equally sensitive to the effects of descriptive social norms. Yet, previous research suggest that norms affect people regardless of their ages,^16^ and teachers/students were randomly assigned to conditions. Another limitation is that the internal reliability for stereotypical ratings towards the East Asian student was minimally acceptable.^43^ Future studies could use other and/or more stereotypical features.

In sum, our study did not find support for the hypothesis that descriptive social norms, stating what the majority thinks or does, has an effect on stereotypical expressions towards medical students with and without migration background. Therefore, anti‐bias intervention do not unintentionally increase stereotypes by communicating a high prevalence of stereotypes. Our study showed that individual preferences and perceptions (i.e., SDO and IWC) rather than the context (i.e., descriptive social norms) were predictive of stereotypical expressions. This implies that an individual preference for group‐based hierarchy and inequality (i.e., SDO) could lead to negative stereotypical ratings towards an individual of a stigmatised group, whereas individual perceptions of working in an inclusive study/work culture (i.e., IWC) could relate to positive stereotypical expressions towards an individual of a non‐stigmatised group.

## FUNDING INFORMATION

Not applicable.

## CONFLICT OF INTEREST

No competing interests.

## ETHICS STATEMENT

This study was approved by an official ethical committee, see manuscript.

## AUTHOR CONTRIBUTIONS

All authors (CvA, MB, WvdB, and KS‐J) were involved in the conception and design of the study. CvA collected and analysed the data. All authors contributed to the interpretation of the data. CvA wrote the first draft of the research paper. All authors contributed to the critical revision of the paper and approved the final manuscript for publication. All authors are accountable for the manuscript.

## Supporting information


**Data S1.** Supporting Information
