# Supplementary material for: Do norms unintentionally increase stereotypical expressions? A randomised controlled trial
Source: Med Educ. 2021 Dec 26;56(3):331–8. doi: 10.1111/medu.14712 (PMC9304281; doi:10.1111/medu.14712)

**SUPPLEMENTARY FILE 1**

***Development of Dependent Measures.*** First, we needed to develop two lists of stereotypes that could serve as our dependent measures. Attendees (*N*= 52) of the Dutch Society for Medical Education conference in November 2019 were approached, including teachers, researchers, medical specialists, internship trainers, educationalists, and policy officers. Attendees were asked two questions: “*Which stereotypes, do you think, prevail in Dutch medical education, with regard to students without (question 1) and with (question 2) migration backgrounds?*” It is unlikely that these answers were influenced by social desirability concerns, as the question focused on what prevails in medical education, and hence, not necessarily their own personal beliefs.

All 104 open answers (52 participants x 2 answers) were coded by two independent raters, who were experienced with qualitative data coding. The raters indicated which student ethnicity group was mentioned, and which features were associated with the particular group. Sometimes different wordings were categorized into the same feature. For instance, linguistically, language proficiency, and communication skills, were roughly ascribed to the feature “communicative”. The frequency of each stereotypical feature was counted, and the common denominators (i.e. shared meanings) were sought in both rater codes. It appeared that students with Asian backgrounds were most often described with very specific features regarding low assertiveness, high intelligence, low communication skills, etc. This was consistent with research showing bias against medical students with Asian backgrounds,^1, 2^ and compatible with descriptive stereotypes that have been found for East Asians in North America.^3^ Therefore, a student with an East Asian background was used as the stigmatized stimulus in our study. A native Dutch student was used as the non-stigmatized stimulus.

Based on the frequency counts in these first findings, four features were selected for the East Asian student (*assertiveness, intelligence, communication skills, and knowledge of Dutch hospital culture*) and five features for the native Dutch student (*ambitiousness, eloquence, competence, diligence,* and *intelligence*). Additionally two lists of all stereotypical features for both the East Asian student and native Dutch student were created (see *Supplementary file 2*).

Please note that features’ scores for the East Asian student stimulus were reversed, because lower ratings indicated higher stereotypical expressions. Yet, for Asian students as a specific group, we should not have reversed the item intelligence, because the findings from the first phase of the study and previous research^2, 3^ (as depicted above) have shown that Asian students are more likely to be stereotypes as high in intelligence, rather than low in intelligence. Our current data (*N*= 158), however, showed that the internal reliability (*ω*) of the features rating would not increase when a) intelligence was treated as a separate factor, and neither when b) intelligence was scored in the opposite direction of the other items. Therefore, we deliberately chose to score intelligence in accordance with the other items, indicating that lower ratings of intelligence equal higher stereotypical ratings for the East Asian student. There are two reasons that could justify this methodological choice. The first reason is that a Halo effect^4^ may have led participants to score intelligence in accordance with the other items (high, high, high, high, or low, low, low, low). The second reason is that the findings from the first phase of the study additionally indicated that students with migration backgrounds, as a general group, are seen as less intelligent. “General” stereotypes about students with migration backgrounds might therefore have contaminated more “specific” stereotypes about East Asian students. Hence, these two reasons could function as reasons as to why it might be more appropriate to consider low intelligence as a stereotype for our East Asian student stimulus in our study, even though this is contrary to what previous research suggests about East Asian stereotypes (i.e. high intelligence).

[1] van Andel CEE, Born MP, Themmen APN, Stegers-Jager KM. Broadly sampled assessment reduces ethnicity-related differences in clinical grades. *Medical Education*. 2019;**53**:264-275.

[2] Yeates P, Woolf K, Benbow E, Davies B, Boohan M, Eva K. A randomised trial of the influence of racial stereotype bias on examiners’ scores, feedback and recollections in undergraduate clinical exams. *BMC Medicine*. 2017;**15**:179.

[3] Berdahl JL, Min J-A. Prescriptive stereotypes and workplace consequences for East Asians in North America. *Cultural Diversity and Ethnic Minority Psychology*. 2012;**18**:141-152.

[4] Lachman SJ, Bass AR. A Direct Study of Halo Effect. *The Journal of Psychology*. 1985;**119**:535-540.

**SUPPLEMENTARY FILE 2**

**Stereotypes East Asian students:**

- Highly intelligent
- Highly intellectual
- Well-educated
- Conscientious
- Not assertive
- Not proactive
- Humble
- Shy
- Quiet
- Not in the foreground
- Nodding “yes”
- Subordinate
- Less communicative
- The urge to prove oneself
- Hard worker
- High performer
- Serious
- Enormously motivated
- Goal oriented
- Oriented towards high grades
- Studying hard
- Strong family pressures
- Parents do not allow failure
- Respectful
- Not reflective
- Not receptive for feedback

**Stereotypes native Dutch students:**

- High social status
- High social milieu
- Highly educated parents, rich parents, rich family
- Arrogant, confident, spoiled
- Parents as doctors, doctors in family
- Status oriented
- Privileged
- Eloquent
- Diligent
- Strong communication skills
- Knowledgeable of Dutch language and culture
- Have a good fit with education system, easily get high grades
- More easily study and find ideal career opportunities
- Normal
- Perfectionistic
- Excel on multiple areas (sports etc.), multi-talented
- Driven
- Successful
- Career oriented
- Ambitious
- Performance oriented
- Oriented toward building CV
- Student organizations, beers and drinks, love to party, have hangovers
- Individualistic
- Stubborn
- Cheeky, direct
- Want to be treated like an equal person
- Strategically study, lax
- Open minded, modern
- Smart
- Competent
- Committed
- Socially skilled
- Autonomous
- Self-aware

**SUPPLEMENTARY FILE 3**

**East Asian student**

**
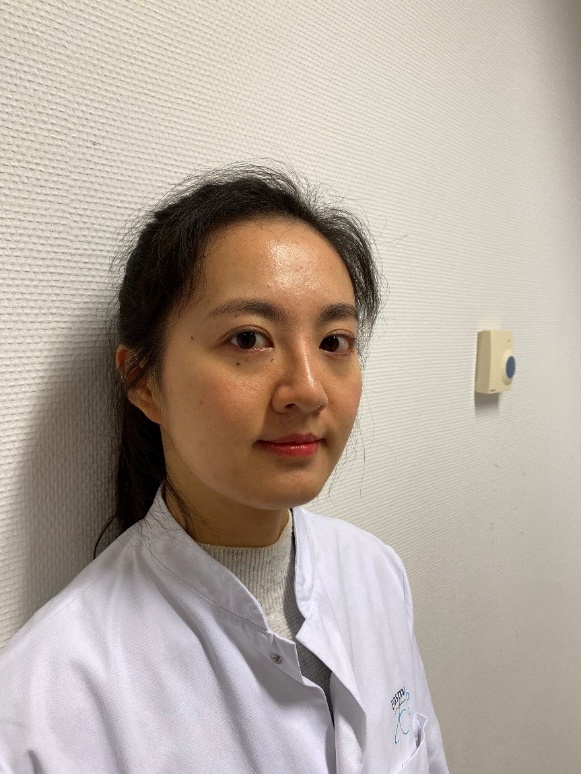
**

**Native Dutch student**


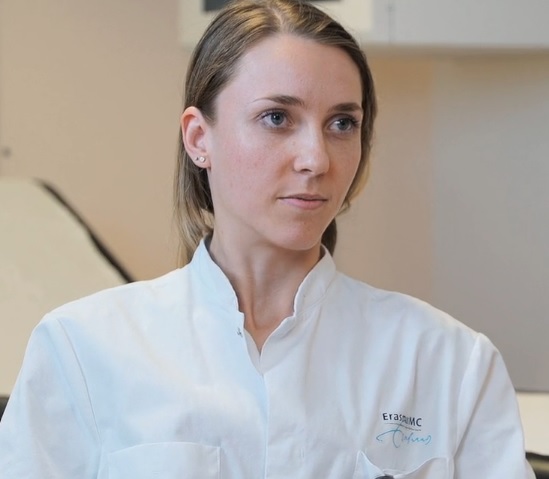

Supplement: Supplementary file 1 — Data S1. Supporting Information [file MEDU-56-331-s001.docx]
